# Case of Ileus

**Published:** 1846-04

**Authors:** 

**Affiliations:** Lemberg


*Case of Ileus; Portion of Intestine expelled by Stool. Recovery.* By Dr. Nagel, of Lemberg.—K. J., a servant, had always enjoyed good health till within the last few years, when he became subject to frequent attacks of colic. On the evening of the 12th Feb., 1843, he was seized with violent pain at the lower part of the abdomen, accompanied with shivering, and frequent vomiting and purging. On admission into the hospital, on the morning of the 13th, he was in the following state: head hot and painful; tongue foul; thirst; abdomen swollen, and tender to the touch; skin dry; pulse full, hard and frequent; vomiting, with watery stools, tinged with blood. (Antiphlogistic treatment.)

The symptoms continued much the same till the 16th, when they diminished in intensity, and the stools were no longer tinged with blood. On the 19th, there was violent tenesmus, accompanied on the 23d with prolapsus of a portion of intestine, which, however, was reduced without causing pain.

On the 26th, the patient free from fever, and altogether in a satisfactory state; passed by stool a portion of intestine, 20 inches long, and at some points 2 inches broad; it consisted of a portion of the ileum, the cœcum, appendix vermiformis, the whole of the ascending arc and a portion of the transverse colon. The mucous membrane was everted, of a brownish color, striated with black, especially at the cœcum; it was soft and easily removed; the peritoneal coat was likewise of a brown color, and corroded, leaving bare the muscular coat, which was also destroyed at some points; for some days after, there was slight pain at the lower part of the abdomen; but on the 23d of March, the patient left the hospital perfectly cured.—*Gazette Medicale, and Edinburg Monthly Journal.—Ibid.*

